# Owners’ everyday interactions with their horse: Pain-related issues and those of veterinary concern

**DOI:** 10.1017/awf.2025.10036

**Published:** 2025-09-17

**Authors:** Rebecca Smith, Liz Perkins, Gina Pinchbeck, Joanne Ireland

**Affiliations:** 1 Department of Equine Clinical Science, Institute of Infection, Veterinary and Ecological Sciences, University of Liverpool, Neston, UK; 2 Department of Primary Care and Mental Health, Institute of Population Health, University of Liverpool, Liverpool, UK; 3 Department of Livestock and One Health, Institute of Infection, Veterinary and Ecological Sciences, University of Liverpool, Neston, UK

**Keywords:** animal welfare, chronic pain, decision-making, equine, ethnography, qualitative

## Abstract

The decisions made by horse owners on behalf of their animal, including decisions to involve a veterinarian, play an important role in the management of pain. This study explored horse owners’ experiences to understand how they conceptualised chronic pain within the context of their horse-human relationship, what led them to seek veterinary involvement, and how veterinary interactions shaped their perceptions of pain and its management. An ethnographic approach using constructivist grounded theory methods was adopted. This paper draws upon field notes generated through 200 h of observation undertaken within four veterinary practices in the UK, as well as interviews with horse owners and carers. Analysis identified that owners’ understandings of pain-related issues of their horse were based upon knowledge of what was normal for their animal, and deviation from this norm. Horse behaviours were ascribed meaning by owners in light of contextual factors, in turn affecting owners’ perceptions of pain. While pain could factor into decisions to initiate a veterinary consultation, it was generally not the specific reason owners presented their animal. Veterinarians’ approaches to identifying and treating painful problems played a role in the formulation of owners’ understanding of their horse’s behaviour. Interactions had implications not only for treatment opportunities, but for perceptions of veterinary expertise. This study highlights the context-specific nature through which pain recognition and decisions regarding a horse’s treatment arise. It highlights the drivers of human decision-making and offers potential avenues to support human behaviour change and improve horse welfare.

## Introduction

Horses and humans live in multispecies societies where animals are reliant upon humans for health care provision, including the management of pain. Pain in animals has been described as “*an aversive sensory and emotional experience representing an awareness by the animal of damage or threat to the integrity of its tissues*” (Molony 1996). Painful issues, such as those involving the musculoskeletal system, are suggested to be widespread across the horse population, and especially in older animals (Egenvall *et al.* 2009; Ireland *et al.* 2012a; van Weeren & Back 2016). Aspects of the horse-human relationship, including a person’s ideas regarding the meaning of an animal’s subjective experience, perceptions of whether or not the animal is in pain, and decisions about if or how to approach pain management, all affect horse welfare. Whilst many people may shape these processes, such as paraprofessionals, peers on livery yards (horse housing premises) or online communities, veterinarians are important due to their legal powers to diagnose, prescribe, treat and conduct certain technical procedures. Therefore, understanding how and why horse owners decide to involve a veterinarian in their animal’s care can assist in identifying ways to support decision-making and optimise pain management practices.

Owners’ reasons to involve a veterinarian in their animal’s care are multifaceted. From owners’ perspectives, the maintenance of horse health and well-being are not exclusively veterinary matters and approaches adopted relate to ideas about how they might aid the horse (Smith *et al.* 2024). An international survey of horse owners suggested that leisure horse owners prioritise quality of care and interpersonal skills when seeking veterinary attention (Elte *et al.* 2024). Animal owners report knowing when a veterinarian is needed even though reasons vary (Owczarczak-Garstecka *et al.* 2022; Muldoon & Williams 2024; Smith *et al.* 2024). Research of owners of older horses suggests that decisions to involve a veterinarian are shaped by an owner’s experiential knowledge of both their horse and of veterinary medicine (Smith *et al.* 2024). In their ethnographic study of horse owners, Jones McVey (2021) discussed two sorts of reasons for seeking veterinary attention relating to concerns about a horse’s behaviour: ‘diagnoses-seeking’ or ‘all clear-seeking’. Therefore, both owners’ everyday relationship with their animal, as well as their expectations or hopes for what expertise a veterinarian might provide, are relevant to the involvement of a veterinarian. Surveys have reported variation in veterinarians’ approaches towards pain management in horses (Price *et al.* 2002; Waran *et al.* 2010; Sellon *et al.* 2022) suggesting factors that are shaping variation in approaches require further exploration.

People’s relationships with animals are dynamic and humans ‘become’ with other species through emergent multi-actor networks (Haraway 2008). As Haraway (2016) writes, “*Natures, cultures, subjects, and objects do not pre-exist their intertwined worldings*”. Therefore, viewing people’s perceptions of an animal’s subjective experience, including pain, as relationally constructed, enables the study of how meaning and action are shaped through everyday connections. Relatedly, the everyday contact and care that takes place over time between owner and horse creates a certain type of knowledge of the individual animal (Smith *et al.* 2022). This knowledge production is also evident in dog-owner relationships (Sanders 1999). In his study, Sanders (1999) described how owners had little doubt of their animals’ cognitive abilities and all could recount examples of what they defined as thoughtful behaviour, and that this was grounded in people’s ongoing and intimate contact with their dogs — it was how they knew their dog. The knowledge of an individual generated through everyday relationships is therefore likely to form a basis for identifying change, and along with the context within which it occurs, frames any involvement of a veterinarian.

Existing evidence demonstrates that many owners consider that horses are able to experience pain. In a Canadian study of equestrian stakeholders, the vast majority of participants strongly believed that horses could experience affective states, particularly pain, fear and boredom (Dubois *et al.* 2018). A horse’s behaviour is described as a means of evaluating whether they are in pain. In a survey of Brazilian horse owners and other caretakers, participants described a variety of body and facial expressions as signs of pain (Hotzel *et al.* 2019). However, pain behaviours are reported to vary between individual animals (Hall & Kay 2024). In horses with chronic back pain, interactions with humans are said to differ with the severity of underlying vertebral problem (Fureix *et al.* 2010). The meaning assigned by owners as to why changes in behaviour occur are likely to be related to context-specific factors. In dogs with chronic pain, for example, owners talked about empathising with their pet owing to their own experiences (Davis *et al.* 2019). Therefore, exploring how owners conceptualise chronic pain within the context of their horse-human relationship is important for understanding how issues become concerns, and the ways in which they may be attributed to pain.

People’s perceptions of equine pain reportedly affect decisions regarding a horse’s management or health care provision. Owners of older horses report that severe or uncontrollable pain in their animal is a factor influencing euthanasia decisions (Ireland *et al.* 2011). However, the attribution of pain can vary, and differences are reported in the identification of painful health conditions by horse owners compared to veterinary examination (Ireland *et al.* 2012b). The presence of abnormal movement or ‘lameness’ in horses is frequently problematised, often with reference to pain as a causal factor. There are concerns regarding the ability of people to recognise movement abnormalities. This not only relates to owners (Ireland *et al.* 2012b; Muller-Quirin *et al.* 2020), but also veterinary students (Starke & May 2017), and practicing veterinarians (Keegan *et al.* 2010). Keegan *et al.* (2010) note that subjective evaluation of lameness by equine veterinarians is especially inconsistent in cases of mild lameness. While assessments of an animal may not always be straightforward, other factors in the veterinary consultation, such as veterinarian-owner communication, are also likely to affect whether and to what extent issues become problematised or examined. In human healthcare, the patient-clinician interaction has been found to shape pain management outcomes. Drawing on the results of their literature review, Henry and Matthias (2018) developed a conceptual model of patient-clinician communication about pain that included stages of information exchange, treatment decision-making, and relational communication. They suggest that factors such as respective understandings of pain, treatment goals, and beliefs about the cause of pain, shaped discussions, but were not always explicitly discussed (Henry & Matthias 2018). However, in the veterinary context, little is known about how consultations shape communication regarding pain or the ways in which this may affect owners’ beliefs or management approaches.

This paper is drawn from an ethnographic study of veterinary practice and focuses upon the owners’ relationships with their horse. The aims of the study were to explore how chronic orthopaedic pain was conceptualised within the broader context of owners’ relationships, to understand the reasons for veterinary advice-seeking, and how veterinary interactions shaped perceptions of pain and its management.

## Materials and methods

### Study outline and ethical considerations

This ethnographic study involved the collection of naturalistic data from four veterinary practices that provided ambulatory services for predominantly leisure (non-professional) horse owners in the UK. Horse owners seeking veterinary involvement for their animal were recruited. The study was approved by the University of Liverpool’s Veterinary Research Ethics Committee (approval number: VREC1281). Participants were provided with an information sheet outlining the purpose of the study, data collection, storage and anonymisation procedures, and time-frame for withdrawal from the study.

The study used an ethnographic methodological approach to study people and animals in a naturalistic setting (Brewer 2000). A relational perspective was drawn upon to examine dynamic processes and relationship networks. This perspective enabled exploration of multiple meanings created through diverse horse-human and veterinarian-owner relationships, and the ways in which these affected decision-making on behalf of the horse. The study drew upon a symbolic interactionist theoretical perspective to consider how understandings of the horse, their health, and subjective experience were made, and how these were shaped by contextual factors. This sociological theory is based upon the premise that individual realities are shaped by experiences and interactions, and that people act based upon meaning and interpretation (Blumer 1969). In line with recent sociological scholarship, we take seriously the significance of animals and their agency in interaction and meaning-making (Arluke *et al.* 2022). However, methodologically, we did not undertake a multispecies ethnography. We did not try to ‘assess’ the animal’s subjective experience — in particular whether they were experiencing pain or not (Hamilton & Taylor 2017).

The constructivist grounded theory approach to data collection and analysis acknowledges the role of the researcher in shaping interactions during data collection, as well as throughout the interpretive process of analysis, see Reflexivity statement (S1; Supplementary material). The substantive theory presented is thus viewed as a reconstruction of this discrete area of social life and one which helps to explain, predict action, and offer avenues for future research (Charmaz 2014).

### Data collection

Veterinary practices were purposively sampled to include a variety of locations, practice size, solely equine and mixed small/farm animal practices, access to hospital facilities, and privately or corporate-owned practices. Practices or gatekeepers were contacted by email, phone call or visit. Potential participants were provided with an information sheet about the study and there was opportunity for discussion with Rebecca Smith (RS). All ethnographic work was undertaken by RS, a female veterinarian who had worked previously as a small companion animal veterinarian and who had experience of caring for, but not owning, horses. RS had completed a PhD and was trained in social research methods.

Data were collected between May 2023–April 2024. The recruitment of veterinary practices continued alongside early data collection. RS spent time in each practice and shadowed veterinarians during selected consultations, through which owners were recruited. Prior to RS’s visit, each veterinary practice was sent a short summary of the study to share with their clients. The text highlighted that some veterinarians would be accompanied by a researcher during the defined time-period. It was at the discretion of the veterinary practice as to if/how they distributed this information. Consultations were selected to be observed based on a range of veterinary characteristics and types of issues, e.g. owner-reported lameness/stiffness/footy/laminitis/foot abscess, owner-reported behavioural issues, repeat prescription examinations (e.g. non-steroidal anti-inflammatory drugs), vaccination, and emergencies (e.g. recumbent horses, non-specific signs such as off-colour). These criteria were chosen based upon the experience of the research team, previous literature indicating that these were instances where chronic pain may be an issue, as well as developing data analysis. Data collection from each practice varied with the practice structure and team, veterinarians’ workload during the period of study (1–2 weeks per practice) and the nature of who booked in for a consultation. Ethnographic interviews with veterinarians took place in the car between visits to gather insight into the context of the veterinarian-owner relationship as well as veterinarians’ experience of the observed consultation and decision-making that took place. Theoretical sampling was adopted and where there were multiple possible consultations that might have been of relevance to the study, developing analysis drove the selection of consultations (see *Data analysis*). In a couple of instances, selected consultations were not attended based upon the veterinarian’s choice due to sensitivity, e.g. euthanasia. Horse owners were recruited based upon the aforementioned factors, and during the consent-taking process requests for a follow-up interview were made.

At the beginning of the consultation all participating owners were provided with a brief overview of the study as well as a participant information sheet and written consent was obtained. Participants involved directly in the observed consultations were asked for informed consent, while others were told about the study more informally and provided with information sheets. Although participants were told that they could withdraw from the study up to two weeks following data collection, none withdrew. Field notes were compiled following consultations, containing observations of conversations, examinations and initial impressions. Time spent in the field involved interacting with and observing additional people, including: reception teams, veterinary nurses, an equine dentist, farriers, yard staff and other owners on livery yards. Time spent at veterinary hospitals allowed for interactions between animal patients, veterinary staff, and owners attending the practice to be observed, and to understand how pain became a matter of veterinary concern within these contexts. Some owners were introduced to RS as they attended the veterinary hospital for diagnostic or surgical procedures following previous consultations, and veterinary team discussion regarding hospitalised patients was observed during daily ‘rounds’ in some hospital contexts. The developing analysis reflected how these multiple interactions shaped RS’s understanding and interpretation of people’s day-to-day lives.

Interviews with owners were held separate to and following veterinary consultations. All owners were happy for the consultation to be observed, and all but one agreed to be contacted subsequently for an interview. Not all participants who agreed to be contacted were finally recruited for interviews. This was due to a lack of response from the owner (following phone call, text or email) and also due to selection of cases by RS based on factors including the perceived relevance of the owners’ experience/consultation to the study aims, theoretical sampling approaches and practicalities of carrying out interviews during fieldwork. Some interviews were held in-person at the horse housing premises, e.g. livery yard or owner’s home, on-site at the veterinary practice due to the owner attending with their horse, or by phone call or video-based platform. In one instance, the owner preferred to answer questions about her experiences via email, and so these data were included in the analysis. Whilst RS sought to contact participants in the day(s) following the consultation to arrange the interview, these were often conducted the following week. The time-frame between the consultation and the interview ranged from immediately following the consultation to five weeks in one case. Interviews were audio-recorded. A semi-structured approach was adopted using an interview topic guide (S2; Supplementary material) developed from previous literature and the authors’ personal experiences of veterinary work. Conversations were centred around the horse(s) attended to during the observed consultation as well as other currently or previously owned horses. Questions varied depending upon the individual’s experiences, and as analysis developed, questions were adjusted. While flexibility in questioning and follow-up based on participants’ experiences is an important approach in ethnographic research, it means that the focus of each interview varied and data were not directly comparable. Participants were provided with a further information sheet that included contact details and sources of support. All interview data were transcribed by a commercial transcription service that uses human transcribers and an intelligent *verbatim* approach. Transcripts were anonymised and pseudonyms are used.

### Data analysis

Data collection and analysis took place side-by-side, based on the principles of grounded theory (Charmaz 2014). Interview and field note data were reviewed concurrently during analysis to add context and enable comparison. An inductive approach to data coding meant that categories and concepts were developed from participants’ experiences, as documented in the interview and field note data. The range of data enabled variation to be explored. Cases or ‘incidents’ were compared to analyse similarities, differences and relationships. Diagramming was used throughout analysis to explore the connection between categories and to explain patterns in the data (Brewer 2000). Early and ongoing analysis enabled theoretical sampling of particular cases that could then be used to ‘test’ and refine categories; for example, an appointment requested for review of two horses kept in a riding centre enabled exploration of the ways in which the horses’ purpose and the type of physical activity required of them affected the construction of issues and related perceptions of pain. The interpretive process of analysis continued throughout the course of writing. Analysis ceased when no new codes were identified and when the categories represented the fullness of the participants’ experiences (Varpio *et al.* 2017).

## Results

In total, around 200 h of overt observation were undertaken including 47 consultations. Semi-structured interviews were held with 25 owners/carers, including one livery owner and one manager of a riding centre (S3, S4; Supplementary material).

This paper discusses three interrelated processes: ‘Owners’ everyday interactions with a horse: Formulating pain-related issues’; ‘Initiating a veterinary consultation’; and ‘(Re)formulating pain-related issues: The role of the veterinarian’. Findings suggest that while pain may be part of an owner’s concern leading to veterinary advice-seeking, it was generally not the specific reason that they presented their animal. Rather, presentation was on account of other matters, such as owner concern about a horse’s health or behaviour, or for preventive health care. These other matters formed the basis of veterinarian-owner interactions and this framing meant that the horse’s subjective experience — including that of pain — was not always explicitly discussed. Owners’ understandings of issues or pain-related issues were reconstructed during veterinary consultations. Veterinarian-owner interactions not only influenced the horse’s access to treatment at the time, but affected owners’ future advice-seeking behaviours, with implications for pain management.

### Owners’ everyday interactions with a horse: Formulating pain-related issues

In their everyday interactions with their horse, owners developed a knowledge of their horse’s body as well as their ‘normal’ behaviour. The context of each relationship and the history of the animal’s health informed the meaning of ‘normal’ or what constituted a deviation from it. Participants talked about changes in their horse’s behaviour, demeanour, or movement, for example, during observations of the horse in the stable, when grooming or tacking up. Many spoke about noticing a change through becoming aware of a sense of ‘reluctance’ of the horse when riding or noticing a decline or difficulty in relation to the horse’s ‘performance’ if involved in sporting activities. Owners used their senses to detect change in the horse, feeling or hearing a change was often reported as an early indication that something was not right:

“*when I was sitting on him, I just felt him dipping down. Not much, but just felt him dipping down maybe*” [Simone, practice A].

“*when I was riding him, you could hear with the leg, which is the near hind, when he was putting it down, when you were walking, it sounded totally different*” [Julie, practice B].

In the context of each relationship, changes in the animal could be characterised as ‘issues’ or ‘health issues’ and these categories formed the basis of any pain-related concerns:

“*So, when you get off you lean forward slightly and then throw your leg over, and he started throwing his head up. That suggests to me that there is something uncomfortable because he didn’t before, he wouldn’t do that*” [Mark, practice C].

Whilst some owners used the word ‘pain’ others spoke about their animal’s comfort, discomfort or being uncomfortable, hurting, aching, or being sore. For some owners, knowledge about pain was based upon interpretations of the horse’s behaviour and feelings:

“*I knew he was in pain, because he had his head down. He was sad*” [Laura, practice A].

Owners came to generate an understanding of their horse’s personality, and this was linked to how they interpreted their horse’s behaviour. In long-term relationships this knowledge could become tacit:

“*I think it’s instinct. Once you know them so well, I think you know when there’s something wrong. Zara, she’s quite independent, she’s a bit bolshy*” [Carol, practice C].

“*To me, certainly, with Tulip, she’s not a nasty horse. Yes, she can be flighty, but she’s not a nasty horse*” [Felicity, practice D].

However, particularly in young animals or newly established relationships, owners could question the intention behind a horse’s behaviour:

“*He’s completely new to us, so it was just trying to gauge, you know, is he in pain, or is he just being naughty?*” [Imogen, practice C].

Owners spoke about receiving opinions or advice from others about the meaning of their horse’s behaviour, be this on livery yards, via online communities, or from paid staff at training yards. The construction of the horse’s personality played a role in this. Some owners acknowledged that their animal’s behaviour could be interpreted in different ways. For Rebecca, whilst she mentions that she could interpret her horse’s behaviour as being confrontational, instead she has learnt that mood and behavioural change are indicative of underlying pain:

“*She’d stumble, and then she’d whip round at you, and, like, you know, clack her teeth at you, as though it was your fault she’d fallen over. Which, obviously, she doesn’t think that, she’s a horse, but she was mad, and it looked like a pain reaction. And that does seem to be her go-to, when she’s sore; she immediately goes to angry. Which, I mean, is helpful, in a way, because at least I know something’s going on*” [Rebecca, practice A].

Personality traits, such as a horse being ‘dramatic’ or ‘stoic’, were also mentioned as factors to consider when deciphering whether a horse was truly in pain. A few participants also raised ideas about horses hiding or masking pain or discomfort, and this was sometimes associated with their ‘prey species’ instinct:

“*But with something that’s longer term, I think they’re absolute masters of deception. It goes back to their prey animal instinct, that they just want to try and hide that they’re the older, sicklier one, or the sore one*” [Rebecca, practice A].

“*I think they do cope with it because they don’t know any better than to cope, and they’re a prey animal* — *they have to cope*” [Marnie, practice D].

While ‘lameness’ was a term often used to signify a (painful) problem, people constructed their understanding of what lameness was and what it meant for the horse in different ways. Knowledge of the horse’s history and their health — such as underlying injury or disease — could factor into how the horse’s movement was assessed. An owner’s understanding of what was ‘normal’ for a horse was therefore related to how a ‘problematic’ gait was defined:

“*So, he wasn’t lame unlevel, but there was that unlevelness, because of that. But then you could justify it, saying that he’d got the muscle wastage, so he was always weaker on that left side*” [Imogen, practice C].

Owners used a range of terms when describing changes in a horse’s movement that might alert one to a problem, for example, stumbling, bent, limp, stiffness, toe dragging, sliding feet, tripping, doddery, short, boxy, and (not) going forward. Associations between the horse’s movement and pain were interpreted in light of the individual horse and their past:

“*But yes, he was always lame, but to me, he wasn’t in pain lame. That was just Pumba’s way of going*” [Felicity, practice D].

In older animals, changes in movement or demeanour — such as one horse who was described as getting grumpy in his old age — were reviewed in the context of what was to be expected as part of the ageing process:

“*We were a bit concerned, but we thought she’d slipped in the mud, because it was so wet and muddy. So, we were worried Sunday night, but we thought, “Well, she’ll probably just walk it off. She’s 26, you know, she gets a little bit stiff*” [Carol, practice C].

Whilst some owners reported identifying what they referred to as lameness, some had doubts about their ability to do so. In one riding centre, the manager reported that due to the slower work required from some horses, staff found it more difficult to spot gait abnormalities. Issues subsequently identified as being of musculoskeletal origin could be raised on account of other behavioural matters. For example, one horse was presented for head-shaking and was subsequently identified as being lame. The head-shaking behaviour was later attributed to underlying pain and visible lameness.

Where issues with their horse had arisen, owners talked about an iterative process of trying to identify the cause in the context of other factors such as the weather/ground conditions, recent events/changes to daily management, the horse’s age or ‘education’ and ideas about their personality. One mare was described as being a ‘bitch’ and ‘sassy’ by her caregivers as they explained grappling with months of unwanted behaviour. These contextual factors were interlinked and shaped the way in which owners attributed meaning, and sought to remedy, issues with their animal:

“*So, his reluctance, you could say it was his lack of fitness, his lack of confidence, lack of education. There was a reason for everything*” [Imogen, practice C].

Husbandry changes were often implemented prior to or following veterinary involvement. Each owner’s context, including their ambitions for the horse and their own lifestyle, shaped the nature and extent to which adjustments to a horse’s management were made:

“*No, he doesn’t want to canter, so asking for canter goes on for half a circle, and then you think, “Oh, right, we’re cantering,” and you sit there and he’s broken. He doesn’t want to canter. I mean, you can make him by ‘boot, boot, boot’ and then you get this feeling like he’s cantering with straight legs. I’ve never had anything like it, to be fair, so it’s just odd. I think I just thought, “I won’t do it. He’s quite happy in trot*”” [Marnie, practice D].

In many cases there was a sense that issues with a horse could multiply over time as numerous changes in the horse were noticed and managed. Owners talked about seeking ways to remedy issues through various means such as doing their own research or involving service providers such as a saddler, physiotherapist, or behaviourist. These people could already be employed on a regular basis or may be sought on an *ad hoc* basis because they were thought to potentially assist with the concern. They played a role in the problem-solving process and could precede, or prompt, the involvement of a veterinarian:

“*So, we had the physio out to him, because he gets physio on a fairly regular basis, because we do expect him to perform, so we’ve got to look after him. The physio noted that he was very sensitive down his left side. He was reacting to that more than he does normally*” [Mark, practice C].

Owners’ knowledge of, and experience with, a particular disease could assist in the identification of the issue in their animal. However, in the face of disease, a horse’s behaviour could change in differing ways and therefore it might not fit with prior beliefs. It was both the perception of the horse’s issue, as well as beliefs about disease, that co-produced meaning. One owner spoke about how the receipt of advice that might have accounted for her horse’s issue had not, at the time, provided a sufficient explanation:

“*Well, before we knew, I asked opinions what it was, and most people said arthritis. And I said, “No, it doesn’t come on that quick*”” [Simone, practice A].

Expectations of typical presentations also informed owners’ understandings about the horse’s subjective experience, and the degree to which they were in pain:

“*She’s never appeared like* — *I’ve seen pictures of horses with lami*[nitis]. *You know, she’s never done the stance, she’s never appeared to be in massive pain. She was a bit… I’d say she was subdued for her, because she’s a very bright energetic pony normally. …She wasn’t doing any kind of stance that appeared like she was in pain. She’s always been eating and things like that so, yeah*” [Olivia, practice C].

A horse’s behaviour and any association with pain was interpreted in light of this complex construction of health and disease. These constructs had individual meaning within horse-human relationships and often differed in meaning from those of veterinarians.

A horse’s response to the use or withdrawal of pain relief informed owners’ understanding of whether the cause of the underlying pain remained:

“*We did have her on Bute for a while, and we could do anything we wanted with her then. Then we took her off Bute and then it came back, very quickly*” [Andrew, practice D].

Having an idea about the cause of a horse’s health issue, whether through a formal diagnostic pathway or not, shaped an owner’s understanding of their horse’s behaviour and how to manage them. In some instances, a horse’s movement could be considered as indicative of them experiencing a degree of pain; however, it was not always clear to what extent ‘abnormalities’ in the functioning of the body were reflective of the horse’s subjective experience, especially in the absence of an abnormal demeanour. There could be uncertainty surrounding how to interpret what the horse was feeling, particularly when signs fluctuated:

“*That’s what I struggle with. There are no obvious clues in his behaviour otherwise that he’s feeling discomfort or pain. It’s just that when you lead him out of the stable first thing in the morning, either to ride or take him to the field, those first steps can be quite short and doddery when he’s feeling uncomfortable*” [Jessica, practice C].

Owners were interpreting their horse’s behaviour from day-to-day and this could fluctuate in line with what, or who, they were interacting with:

“*Some days he has good days; some days he has not so good days when he’s really lame; and then other days, he can be flying around the field like something stupid. You know what I mean? When the ground is like it is now, he’s a lot better… Okay, when it’s soft, yes? Yes, and I can reduce his Bute. I mean, for a horse that size, he has two Bute a day, but when it’s like this, if he’s coming in alright and what have you, I won’t give him one. You know what I mean? You know what I’m saying? I don’t give him it just for the sake of giving him it. I mean, he’s been on Bute now for about the last 10 years*” [Betty, practice C].

These daily assessments — sometimes documented in written diaries — could be compared to produce an understanding of pain:

“*I kept a diary of how he was, you know, day-to-day, because some days could be better and you just live day-to-day by, “Oh, he’s a bit brighter. Oh, he’s not lying down as much,” because he was lying down a lot and he was on Bute. He was in pain*” [Pat, practice C].

An owner’s perception of their horse’s degree of pain and ability to cope was informed by the horse’s behaviour. This shaped attitudes and approaches to the use of pain-relieving medication. Past experiences with other horses under their care also played a role. In addition, some owners talked about their own personal pain management approaches — for example, the use of massage, magnetic bands, or oral analgesic medication — when discussing their approaches as regards to their horse:

“*my theory is if it helps him and keeps him going, then I’m all for it. I’m a big believer in Bute. I know a lot of people don’t like it, but my previous horse, he’d have been in a tin if it wasn’t for Bute. That kept him going. And I’m a big pain… I pop a pill for anything. So, I’m a bit the same with him*” [Julie, practice B].

When discussing pain-relieving medication a few owners raised concerns during consultations, or in interviews, about the long-term use of non-steroidal anti-inflammatory drugs in horses:

“*And also, I do think, you know, in terms of protecting them long term, I’ve always been told, you know, don’t give too much Bute, it’s not great for their liver, long term, and stuff like that*” [Maisie, practice D].

Therefore, personal experiences, beliefs about benefits or harms, and in some cases veterinary direction, all shaped decisions regarding whether to use or adjust the administration of pain-relieving medication:

“*I don’t even like giving the kids medicine…I don’t like giving meds. I don’t ever take medicine. But with Star I can see when I can stop*” [Debbie, practice A].

Sometimes pain relief could only be accessed by owners from a veterinarian and this could drive veterinary involvement but, in many cases, especially with short-term use, owners used doses left over from previous prescriptions or obtained medications from friends. There were examples of the use of a ‘Bute trial’ initiated by owners preceding the veterinary consultation. However, in some cases, owners perceived it necessary to withhold any pain relief to avoid ‘masking’ the pain and facilitate a veterinary assessment, with one owner reporting being previously advised this by a veterinarian.

### Initiating a veterinary consultation

Owners utilised veterinary services in a range of contexts including advice-seeking in instances where previous attempts to remedy issues with a horse’s health or behaviour had not been entirely successful, due to sudden change in the horse, or for preventive health care or treatment such as vaccination or dentistry. The services of a veterinarian were sought at a time where an owner wanted to access veterinary expertise, investigations, procedures or prescription medications under veterinary control, e.g. pain relief. Multiple issues in relation to an individual horse or a number of horses could be combined into one consultation. Horses with ongoing health issues could also see a veterinarian for a ‘new’ issue that had arisen.

The initiation of a veterinary consultation could be prompted by interactions within the setting in which the horse lived. This could variably involve the horse’s owner, friends, family, livery staff, trainers or competition judges. Different parties and their interaction could prompt the involvement of a veterinarian by the owner:

“*I think she has always been a little bit reluctant, I suppose, to go forward, in some ways. I think a little bit. But never to the degree of being really concerned about it. Young horse, lots of time to grow, etc. And then Izzie, on that Friday…she got on her and rode her. I wasn’t there, and she said to me afterwards, “I think we need to get someone to look at her because she’s just not going forward, just not comfortable*”” [Heather, practice D].

While horses could be presented for a range of issues, pain *per se*, did not appear to be the driver of advice-seeking behaviours. The presentation of the animal was often on account of other matters relating to issues of the horse’s behaviour or movement:

“*The owner reported that her daughter rides the horse and had noticed him being tense and ‘sticky’ when riding, the mother reported small bucks* — *perceived to be different from ‘excitable’ ones he normally does after each jump* — *which all indicated to them that there was a problem requiring veterinary attention*” [Field notes practice C].

In a few instances, following RS’s introduction at the start of the consultation, the owner responded by raising the issue of pain as part of their concern and reason for calling the veterinarian. Pain as a factor driving advice-seeking was also expressed during an interview with one owner:

“*Yes, because if we can find out what is causing that and stop it, then they will remove that area of pain for him*” [Mark, practice C].

Where a horse had a previously diagnosed health condition, there were cases where owners spoke about a recognised change indicative of pain that prompted veterinary involvement:

“*When I felt him being stiff and sore again, I’d then come back and have him re-medicated*” [Julie, practice B].

Consultations relating to ongoing prescription medication could be combined with preventive health care measures. These consultations could become opportunities for discussion about the horse’s health and pain management, with issues being raised or reassurance sought by owners:

“*that visit was just a touching base one, to get more bloods taken, so we could check his levels, and for* [veterinarian] *to have a look at him and make sure she was happy with how he was on the Bute*” [Jessica, practice C].

While these were often classed as ‘routine’ consultations, expectations stemming from an owner’s life with their horse framed the consultation and concerns relevant to pain were sometimes raised. During a consultation for a 30 year old pony that had been booked into the veterinary diary for blood tests as part of treatment monitoring, the owner raised a degree of concern and uncertainty about her pony’s comfort in light of his age and the fact that a previous veterinarian had discussed underlying knee arthritis. While some discussion with the veterinarian ensued — during which the owner spoke about her observations of the pony running around the field and that he seemed happy doing that — in light of observed evidence of the pony quidding (i.e. dropping balls of partially chewed feed, typically hay, from the mouth), this concern was prioritised by the veterinarian over other less ‘immediate’ issues, and a plan made for a dental assessment. It was common that where multiple concerns about a horse were raised some were prioritised over others. While a follow-up veterinary consultation was planned, it was mentioned that this would be with another veterinary colleague, and thus the veterinarian-owner relationship as well as the framing of the subsequent visit would differ.

A veterinarian’s involvement in issues of horse health or behaviour were generally preceded by other avenues. Going down the ‘veterinary route’ was not the first course of action sought:

“*I couldn’t work out what was causing it and as we’d excluded obvious things like teeth, back, feeding, muscling up and stuff, that it was still happening very intermittently. I felt that I’d done what I could as a non-vet person to rule out what was going on*” [Mariana, practice B].

For some owners, particularly those of younger animals or horses involved in competitive sport, their hopes and expectations of the veterinary consultation were intertwined with the horse’s functionality and performance. Some owners sought a diagnosis and ideas about possible underlying health issues influenced presentation to, and the requests of, the veterinarian:

“*So, after that first time when I came off, so then I just thought… I had him scoped*” [Imogen, practice C].

An owner’s perception of the issue at hand as well as past interactions with veterinarians played a role in how they went about initiating a veterinary consultation. There was evidence of attempts to maintain a certain degree of control over what went on, for example, some owners chose to speak to the veterinarian beforehand:

“*So, I had a conversation with* [veterinarian] *as well, before she came, saying, “I know whatever there is, we’ll need to do something about it if needs be, but can we also consider the fact that she’s a young horse? I’m willing to put her out for six months if that’s what it takes, put her on hold, if it’s just a case that she needs more time to grow or that she just needs more time to develop*”” [Sarah, practice D].

While sudden change in an animal could prompt veterinary involvement, it was still common that other sources of advice were sought in advance. One owner, Grace, had recently bought an older horse and upon decline in the horse’s condition she consulted the horse’s previous owner. Together they made the decision that it would be best to euthanise the horse and a veterinary consultation was booked. However, for Grace, this visit was framed in light of extensive veterinary investigations undertaken with a previous horse that had (from her perspective) been unsatisfactory. Therefore, whilst she requested the services of a veterinarian, the consultation was imbued with a sense of anxiety:

“*I was concerned that he was going to try and convince me to try more painkillers for longer, and I didn’t want that. I knew I was doing the right thing for Lana before she started struggling. So that was a massive thing because I was really, really anxious that he was going to try and persuade me to go more down the veterinary route. I’m really glad he didn’t because that would have been the worst thing for me on Monday*” [Grace, practice C].

Some owners spoke about differentiating between veterinary practices based upon the type of health care measure, or horse health issue, that they perceived to require veterinary involvement. For a few owners this meant that when less ‘specialised’ services, such as vaccinations were needed, convenience in accessing veterinary services was given priority over expertise or access to specific facilities. Being able to request a particular veterinarian was important for some owners. Choices were based upon factors such as previous positive (or negative) interactions with a particular veterinarian or beliefs about the particular expertise of one veterinarian in relation to the issue at hand:

“*If ever he had laminitis again, I would request* [original veterinarian]. *If it was something serious, I might request some that I want more than others. Probably* [original veterinarian]*, because I like the way he deals with things. But for something routine, it doesn’t matter, does it? It’s just whoever comes. I’m not going to start requesting* [original veterinarian] *because he might be off, it might be inconvenient that they send him, and stuff like that. So, there’s no point in doing that, is there?*” [Jennifer, practice A].

However, requests were not always made, or possible, and one owner did not want to inconvenience the practice by doing so unless it was ‘necessary’:

“*Yes, I think, but it’s hard to say, “I don’t want so-and-so,” or whatever. Yeah. I think the only time I’ve actually done that was the visit afterwards. I didn’t want to recall the same vet*” [Pat, practice C].

Therefore, an owner’s understanding of the horse’s issue shaped the nature and timing of presentation to a veterinarian. Pain was always an issue related to other matters. Past experiences with veterinarians informed owners’ understanding of what the interaction might entail and influenced any involvement in their animal’s care.

### (Re)formulating pain-related issues: The role of the veterinarian

Veterinary consultations informed owners’ understanding of issues and whether or not these were indicative of equine pain. For one owner, Mariana, subsequent to previous management adjustments and veterinary visits, eventual advice from a veterinarian she knew led to her reformulate how she understood her horse:

“*And now I know what I know about him, I think a lot of his behaviour was linked with pain and discomfort rather than being a pain in the neck buzzy horse, if that makes sense?*” [Mariana, practice B].

Veterinarians’ interactions with owners informed understandings about how to manage painful problems. One owner, Jennifer, spoke about receiving veterinary advice when managing her horse with chronic laminitis:

“[Veterinarian] *said, I mean, ideally you’d want them in a winch off their feet, that would be the ideal, and then their feet could heal and you’ve not got that weight pressing down. So, for me, when I came and Otis was still lying down and he’d have his breakfast lying down* — *absolutely fine. So, if he wanted to lie down, it was great*” [Jennifer, practice A].

The meaning ascribed to her horse’s behaviour was interpreted in light of this information. As the following quote highlights, Jennifer also understood her horse to have a degree of responsibility in the management of his own pain:

“*We were limiting it to a certain point but there’s only so much you can do for that, and the rest, he has to* — *not just get on with, but in his head, he has to manage it himself. So, coming off his feet a lot, that helped him. So, he did that, he would lie down a lot. That’s him managing his own pain, isn’t it? We’re doing what we can, we’re not just leaving him to it. We’re doing what we can, giving him a nice bed, letting him do whatever he wants to do, keeping him confined and everything else. And then he’s doing his part, he’s keeping off his feet as much as he can and he’s relaxed*” [Jennifer, practice A].

The consideration or use of pain-relieving medication was common in the study, with the benefits or harms discussed in a variety of consultation types. Importantly, the nature of the veterinarians’ handling of queries or concerns informed owners’ perspectives:

“*The veterinarian advised the owner to continue non-steroidal anti-inflammatory drug use and the owner asked whether it was better to dose every other day or to use half a sachet daily. The veterinarian said to use every other day as the drug stays in the ‘system’. The owner then asked, “You shouldn’t keep them on Bute long term, should you?” and the veterinarian said “Well, at 21 years old* [pause]*”, inferring that it was acceptable to do so*” [Field notes practice C].

In the interview that followed, the owner mentioned her concerns and that these had been raised by looking on websites and social media, including Facebook. She reflected on her veterinarian’s response to these concerns and the way that the horse’s age played into this:

“*Well, it’s long-term effects on the liver and kidneys, and of using anti-inflammatories. You have to consider that. Given his age, I wasn’t too worried, but I just sort of wanted to double-check with* [veterinarian], *again, for peace of mind, what her feelings were. She just sort of echoed what I was thinking. “Well, you know, he’s 21*”” [Jessica, practice C].

While the owner appeared satisfied with the veterinarian’s response, an in-depth discussion about the owner’s concerns or ways of approaching pain management as the animal aged did not take place.

Owners’ level of satisfaction of veterinary services was shaped by the extent to which they felt listened to by the veterinarian and the nature of examinations of the horse, both of which informed the perceived relevance and uptake of veterinary advice. While the horse was the focus of veterinary care, its navigation was embedded within relationships. As Marnie highlights below, veterinary advice could also have implications for how owners viewed themselves and their role in the horse-human relationship:

“*He just said, “There’s nothing wrong with him. He’s dead sound,” so you think, “Oh, it is me?”*” [Marnie, practice D].

This example demonstrates the centrality of the horse-human relationship from the owners’ perspective. It also highlights the focus on the horse being ‘sound’ as an indicator of wellness in the veterinary context. In this case, following a period of time where ridden issues did not resolve, Marnie went on to seek further investigations and a different veterinarian later diagnosed her horse with a painful orthopaedic condition. At the time of the consultation being referred to, Marnie’s decision-making was nevertheless informed by the role of the veterinarian and her reformulation of pain-related issues.

Differing views between an owner and veterinarian regarding the presence or nature of a horse’s issue, or a lack of clarity from veterinarians regarding its cause, left lasting impressions on owners. The perceived accuracy or usefulness of veterinary advice was reviewed by owners in light of how the horse’s issue subsequently evolved. Therefore, the provision of veterinary services during each interaction had implications for the horse’s access to pain management as well as future veterinary involvement:

“*They would say, “Well, you know, he’s old.” They wouldn’t give… They’ve got a problem with giving you a definitive answer, which is quite frustrating. So, I got the old vet who used to look after him because he was at* [veterinary practice] *he’s a very good vet*” [Andrea, practice D].

Where veterinarians were involved in an animal’s care, veterinarians’ attitudes and approaches shaped whether painful issues were identified or treatment decisions ensued. As pain was always a concern related to other matters, perceptions of pain and approaches to management were affected by how these other matters were handled.

## Discussion

Owners could construct pain as part of a concern prior to, or as part of, any veterinary involvement. Attempts to remedy issues with a horse’s health or behaviour could drive veterinary advice-seeking behaviours, however, this was not always the first course of action adopted by the owner. The individual horse-human relationship and its history framed the veterinary consultation from the owner’s perspective. The degree to which an owner’s reason for seeking veterinary involvement was addressed by the veterinarian impacted on the degree of satisfaction experienced by the owner. The veterinary consultation shaped the meaning of the horse-human relationship for the owner, in some cases, redefining the meaning of the horse’s health or subjective experience. This could also inform owners’ ideas about appropriate ways to manage their animal. The handling of issues related to the horse within consultations shaped the horse’s access to treatment both in the short, and longer term.

The specific ways in which humans and horses came to relate to one another — to affect and be affected by (Despret 2004) — constituted the (re)creation of meaning. Owners’ understanding of their animal’s behaviour and personality played a part in how issues were perceived and managed and affected the timing of veterinary involvement. While knowledge of the individual was the basis for owner concerns, factors such as the horse’s age also commonly shaped the meaning attributed to horse behaviour. Discourses shape expectations of what is normal or abnormal and thus shape the understanding of pain-related behaviour. In this study, it was common for younger and older animals to be attributed traits associated with construction of the human life course. Historical changes in language use associated with older people, such as ‘elderly’ and other synonyms, have become increasing associated with negative stereotypes, particularly those related to physical health and illness (Ng *et al.* 2015). The World Health Organisation (2025) highlights issues related to ageism across society: “*Ageism affects how we think, feel and act towards others and ourselves based on age. It imposes powerful barriers to the development of good policies and programmes for older and younger people and has profound negative consequences on older adults’ health and well-being.*” These societal issues also appear to be shaping the leisure horse sector and suggest a role for future widespread campaigns (Officer *et al.* 2016). Drives to improve horse welfare might focus on encouraging reflection at an individual, societal, and professional level about what ‘normal’ behaviour or ‘acceptable’ treatment is for horses across age groups to ensure that individualised care is best realised.

The study indicates that some caregivers may place a degree of responsibility on the horse to play a role in managing their own pain. Putting responsibility onto the animal — especially in a context where their level of agency is limited — is likely to be associated with negative welfare consequences. Furthermore, where behaviours indicative of pain are deemed ‘normal’, or the inference made that the animal is choosing to behave in a particular way, this may hinder effective pain management. A survey of UK equestrians noted possible associations between respondents blaming the horse for displaying problematic behaviour, the use of derogatory terms to describe problematic equine behaviour, and use of punishment in response to these behaviours (Girgis *et al.* 2025). Ensuring that effective action to accommodate a horse’s needs is adopted by owners may require shifting discourses, and in the case of pain management, better support for horse owners in terms of practically managing and balancing health and welfare needs. This is likely to require collaboration between stakeholders across the equestrian industry, from individual horse and livery yard owners to paraprofessionals as well as veterinarians.

The social and environmental context of horse-keeping was intimately related to the owner and their own sense of identity, and this had implications for pain management. Jones McVey’s (2021) ethnographic study of horse owners highlights that a horse’s behaviour could be accounted for in different ways by different people, even in the context of a veterinary diagnosis. Social dynamics and the extent to which the behavioural issues were attributed to the owner’s ability or to the horse and their health, was argued to have the potential to delay ‘diagnosis-seeking’; for example, if perceived to reflect negatively on an owner’s confidence or skill (Jones McVey 2021). Our study shows that a number of people or professional groups might be involved in a horse’s management across their lifetime, and all could play a role in reconstructing the meaning of horse behaviour. Encouraging reflection about what individual animals might be experiencing whilst considering how this might be perceived by the owner will be important in supporting pain conversations. Furthermore, our study shows evidence of owners’ own personal attitudes towards pain management influencing approaches in their animal. Strategies to improve pain management in horses will require acknowledgement of the entanglements of human and animal health and the ways in which personal and cultural contexts shape decision-making for animals.

While chronic pain can be defined as pain that persists beyond the healing phase (Apkarian *et al.* 2009), in our study there was no clear-cut time-frame in which issues were constructed as being painful, or upon which intermittent or longer-term issues became ‘chronic’. Even with diagnosed chronic health conditions, fluctuations in a horse’s condition occurred over time. It seems that (chronic) pain could be or become part of an owner’s concern, and the horse was often presented to a veterinarian on account of other matters. In human healthcare, it is reported that body changes resulting from illness are often challenging to differentiate from everyday life, particularly in the early stages of disease. A study of glaucoma patients reported that even when people experienced changes in vision these were difficult to distinguish from minor problems that afflict most people from time-to-time and were perceived to be related to ageing or otherwise expected deterioration (Green *et al.* 2002). Furthermore, patients with concurrent eye problems found it particularly hard to distinguish change that was specifically related to glaucoma (Green *et al.* 2002). While the identification and treatment of pain is important to reduce the risk of pain chronification, findings suggest that pain recognition is not always easy in the context of everyday life. There may be a role to play in increasing education of equine (pain) behaviour to improve treatment seeking. In a small study of dog owners, exposure to educational material regarding signs and symptoms of canine pain using a survey methodology, resulted in significant increases in reported concern regarding the observed behaviour and related intention to seek veterinary care (Kogan 2024). As animal welfare is closely linked to emotion, it is encouraging that research to identify behaviours associated with positive and negative emotional states is ongoing (Phelipon *et al.* 2025). However, our study demonstrates the need for any educational campaigns to emphasise the individual, dynamic and subtle nature of behavioural change that may be observed by owners. Furthermore, attempts to build veterinarian-owner relationships must consider how decisions to involve a veterinarian in an animal’s care are multifactorial and extend beyond understanding of the horse’s immediate issue.

Owners generally viewed pain as indicative of something requiring a veterinary consultation, but other management strategies or forms of advice often preceded veterinary involvement. Owners’ perceptions of a painful issue and ‘appropriate’ ways to manage a horse were shaped by the information provided by veterinarians. Notions of the horse’s own responsibility over pain management reflects self-care discourse in human healthcare literature and may act as a barrier to care (Grady & Gough 2014). In our study, one owner talked about reframing her horse’s issue in light of veterinary assessment leading her to consider whether she was the cause of the issue, instead of the horse’s health. Therefore, interactions with veterinarians have the potential to influence an owner’s sense of self, and in turn this has implications for how issues of the horse may be (re)presented for veterinary attention. Whilst theories have sought to account for patient health behaviours, for example, the health belief or social inequalities model (Dahlgren & Whitehead 2007, 2021; Gilson *et al.* 2011), in the veterinary context there is increasing attention to the role of veterinarians in shaping owners’ approach to animal care including the (re)presentation of their animal (Smith *et al.* 2024, 2025). Findings also suggest that veterinarians’ attitudes to older animals may limit in-depth discussion about the individual’s subjective experience, including that of pain.

Viewing horses as prey species played a role in the normalisation of behaviour, and in some cases, this ‘fact’ was considered to complicate the interpretation of a horse’s subjective experience. Nevertheless, owners generally relied upon informal and embodied ways of recognising change in their animal, and through this, developed individual ways of identifying change that could lead to veterinary advice-seeking. While increased knowledge about behavioural indicators of pain may broaden categorisations of ‘pain-related issues’, for example, through the use of frameworks such as the ridden horse pain ethogram (Dyson 2022) or pain assessment scales (van Loon & Macri 2021; Auer *et al.* 2024; Howard *et al.* 2024), uptake of formalised measures may not fit with owners’ everyday care practices. In light of the variation in how horse behaviour might change when they are experiencing pain, recognition and differentiation of pain from other emotional states is likely to benefit from familiarity with the individual animal (Hall & Kay 2024). In parent-child relationships, the parents’ knowledge of their child is important in terms of pain assessment, however parents may represent this knowledge about their child being in pain in different ways to medical practitioners (Loopstra *et al.* 2015; WellChild 2024). In human healthcare, interdisciplinary approaches to studying chronic pain have been fruitful in expanding debate and offering ways of representing and communicating complex sensory and emotional experiences (Padfield & Zakrzewska 2021). As people construct animal pain within the context of related concerns, it may be necessary to consider, and legitimise in clinical encounters, the multitude of senses and types of knowledges that owners develop about issues in their horse. Furthermore, a focus on relational aspects of communication, allowing owners’ wishes or concerns to be addressed, will also be important in supporting pain management conversations.

This study has some limitations. Participants were recruited via appointments booked in at the veterinary practice. Whilst purposive and theoretical sampling were utilised, the ability to select owners with certain characteristics was restricted and dependent upon people utilising veterinary services during the study period. The reported reason for one owner declining to be contacted later for an interview was due to time commitments. Therefore, relevant case examples may not have been captured. Nevertheless, qualitative research does not seek to obtain representative samples, rather the aim is to generate in-depth understanding (Green & Thorogood 2018). The recruitment of owners using face-to-face methods enabled engagement with communities that may not otherwise take part in research. RS met participating owners through introduction by their veterinarian, and while this introduction facilitated rapport building, it may have introduced some power dynamics. Whilst confidentiality was stated, disclosure by owners may have been limited due to this association with a person/group that was involved in treating their animal. The time lapse between the consultation and the interview may have led to details being lost or altered and introduced possibility for new information to shed light on the issue. Interviews were arranged at owners’ convenience, and many opted to use remote methods, which may have affected rapport building and the depth of data obtained. Nevertheless, owners were often busy and had already had to schedule the veterinary consultation alongside their other commitments. The lack of time spent with the participant in their everyday setting limited observations that may have contributed depth to the study. Alternative approaches to data collection, such as the researcher travelling separately to the veterinarian and thus being able to interview the owner immediately following the consultation, were considered. However, this would have introduced limitations with regards to collecting data from veterinarians during travel periods and the owner introduction made by the veterinarian.

### Animal welfare implications

This study highlights how relationships and context affect the meaning assigned to a horse’s behaviour, and their subjective experience. It demonstrates numerous ways in which individuals, communities, and professionals shape pain management outcomes; highlighting how pain recognition and management is not an individual endeavour. Whilst understandings of the relationship between the horse’s ‘issue’ and pain shaped decision-making, interpretations were often implicit in the veterinary consultation. Whilst theoretically denoting pain, movement abnormalities or ‘lameness’ were often the focus of concern. This may be a barrier to pain management that is of relevance to the wider animal welfare community. This study highlights that veterinarians should be cognisant of the ranging presentations where pain may be an underlying factor. As the (re)presentation of a horse to the veterinarian hinges upon their owner, attention to relational communication approaches that enable owners’ knowledge or concerns to be raised as well as a focus on establishing individualised monitoring approaches will be of ongoing importance.

## Conclusion

Owners’ perceptions of pain are context-specific and rooted in relationships. As perceptions of pain emanate from the horse-human relationship these may differ from that of veterinarians. Concerns about pain may present to a veterinarian in a range of instances. Veterinarian-owner interactions do not necessarily provide opportunity for in-depth discussions about the horse’s behaviour or interpretations of their subjective experience. This has implications not only for treatment opportunities, but for owners’ perceptions of veterinary expertise and their future advice-seeking behaviours. This study suggests that campaigns seeking to improve pain management in practice will need to consider the iterative nature of how pain is conceptualised within particular horse-human relationships. Attention to the matters surrounding pain management will be needed to ensure effective behaviour change strategies that support animal welfare.

## Supporting information

Smith et al. supplementary material Smith et al. supplementary material

## Supplementary material

The supplementary material for this article can be found at http://doi.org/10.1017/awf.2025.10036.
