# Supplementary material for: Owners’ everyday interactions with their horse: Pain-related issues and those of veterinary concern
Source: Anim Welf. 2025 Sep 17;34:e62. doi: 10.1017/awf.2025.10036 (PMC12451390; doi:10.1017/awf.2025.10036)
Supplement: Smith et al. supplementary material [file S0962728625100365sup001.pdf]

Owners' everyday interactions with their horse: Pain-related issues and those of veterinary concern

Rebecca Smith[1]https://orcid.org/0009-0007-2685-728X, Liz Perkins[2], Gina Pinchbeck[3], Joanne Ireland[1] https://orcid.org/0000-0002-5737-1502

[1] Department of Equine Clinical Science, Institute of Infection, Veterinary and Ecological Sciences, University of Liverpool, Neston, UK

[2] Department of Primary Care and Mental Health, Institute of Population Health, University of Liverpool, Liverpool, UK

[3] Department of Livestock and One Health, Institute of Infection, Veterinary and Ecological Sciences, University of Liverpool, Neston, UK

Author for correspondence: Rebecca Smith, email: rebecca.smith@liverpool.ac.uk

Supplementary material

S1. Reflexivity statement

The position of RS as a veterinarian facilitated access to the field sites but also entailed ongoing navigation of this role and the power dynamics that came with it, particularly those associated with entering the world of horse owners via their veterinary service provider. Being a relative outsider – a non-horse owner and non-equine veterinarian – made it more socially acceptable to be curious about and question participants' taken for granted assumptions about the world. However, such experiences may have blinded RS to certain aspects of participants' realities. As an example, during early data collection, informed by scientific training and concepts, questions were framed by asking what participants thought chronic pain was. Participants often found this challenging to answer and responded with reference to how they would know if there was something wrong with their horse. Upon reflection, RS began to consider that this may be related to how owners understood their own experiences and generated knowledge of their animal, and considered this crucial to understanding how their perceptions of pain were constructed. As such, questions were

iteratively rephrased to explore this further. Over the course of the study, this ongoing reflection and questioning was essential to ensure that the study remained grounded in participants' experiences.

S2. Interview topic guides

Veterinary surgeon

Experiences

- Could you tell me about your background working as a vet?
- What is your caseload like? (Prompts: What is the setup of the practice? Are clients able to contact you directly or via a reception team? Do you see your clients regularly for routine and emergency visits? How do you manage on-call?)

Pain

- How would you know if a horse had orthopaedic-related pain? (Prompts: What do you rely on to assess a horse's pain? Do you use any particular scales/frameworks? Would this differ in certain cases? What is the owner's role?)
- Are there any cases we've seen since I've been with you where you feel that a horse has had this type of pain? (Prompts: How did you feel about the outcome of that visit? How do you feel about the conversations that took place between you and the owner? Were you able to discuss your concerns about the horse with the owner? Did you have

any concerns about what was discussed e.g. possible diagnosis/investigations/treatment plans?)

- What does chronic pain mean to you? (Prompts: How would you know if a horse had chronic pain?)
- What are the most common orthopaedic-related cases that you see? (Prompts: Are these chronic cases? What are your main concerns in these types of cases? Are these the same concerns as owners have? Do you have any difficulties in assessing a horse's pain? How do you approach conversations about chronic pain?)
- How would you go about managing chronic pain? (Prompts: What have you learnt is successful or unsuccessful? What do you feel about the treatment options you have available to you? What would prompt you to approach these cases in a particular way? Would any factors limit your ability to manage a horse successfully?)
- How would you go about monitoring a horse's level of pain? (Prompts: Do you think that your approaches differ in any way from an owner? How might monitoring be improved? How are clinical records used to do this?)
- Are there any instances where you think that chronic pain is necessary or acceptable? (Prompts: What are these contexts? How might you go about resolving such issues?)

Quality of life and euthanasia

- What do you think about chronic pain in relation to quality of life? (Prompts: Has your understanding of chronic pain has changed over time? What has changed your views? Does this make you act any differently? Do you have any regrets about how you have managed previous cases?)
- How are these beliefs factored into your clinical decision-making? (Prompts: Are there any differences in how you and your colleagues make quality of life judgements? Has this caused any conflict?)
- How are these beliefs factored into advising owners about euthanasia?

Closing questions

- Is there anything that you might not have thought about before that has occurred to you during this interview?
- Is there anything else you would like to add?
- Is there anything you would like to ask me?

Horse owners

Scene setting/experience

- Can you tell me about you and your horse?
- Could you tell me about your reason for booking the vet visit that I attended? (Prompts: Follow-up on specific points if mentioned e.g. signs of first noticing a problem, other strategies/advice sought in attempts to remedy the problem. If it was a routine visit, ask what they had hoped to get from the visit)

Reference to observed consultation

- How do you feel about the outcome of the consultation? (Prompts: Were you able to discuss any concerns you had about your horse with the vet? How do you feel about

the conversations that took place between yourself and the vet? Did you have any concerns about what was suggested e.g. possible diagnosis/investigations/treatment plans?)

- What is your plan for managing the problem going forward?
- How do you see the role of the vet in your horse's care? (Prompts: What kind of relationship do you have with the vet? Have you seen this particular vet previously?)

Management & care

- Could you describe any accommodations you have made for your horse? (Prompts: What made you decide to change your management practice? How do you feel that has gone?)
- Have you ever had a concern about your horse's behaviour? If so, how did you try to resolve it? (Prompts: Have you changed your approaches over time? Where did/would you go to for advice?)

Pain

- How would you know if your horse was in pain? (Prompts: What do you think is important to this process? Do you think particular people are better at this than others? Do you have any difficulties in deciphering if your horse is in pain?)
- How do you know where the source of the pain is? (Prompts: How would you go about finding out?)
- If you thought your horse was in pain, what would you do? (Prompts: If use of NSAIDs is mentioned, how are these obtained? If employing a physiotherapist or chiropracter etc., how did you decide who to employ?)
- What does 'long-term' pain mean to you? Is this different in any way from 'chronic' pain?
- Have you ever thought that someone else's horse was in long-term pain? (Prompts: What do you think this means for the horse? Are there any occasions where long-term pain might be acceptable for a horse?)
- If you though your horse was in long-term pain would this make you think any differently about how you would manage them? (Prompts: Would it make you think differently about their quality of life? What about in relation to end-of-life decision-making?)

Closing questions

- Is there anything that you might not have thought about before that has occurred to you during this interview?
- Is there anything else you would like to add?
- Is there anything you would like to ask me?

S3. Veterinary practice details

Practice site identifier	Practice details	Approximate hours of observation	Consultations observed	Informal/ethnographic veterinarian interviews	Recorded semi-structured owner interviews
A	RCVS accredited Equine General Practice (University)	27	9	2 (1 female, 1 male)<br><br>Employed veterinarians, between 8 and over 25 years graduated.	4
B	Corporate-owned, RCVS accredited Equine Veterinary Hospital	53	14	11 (8 female, 3 male)<br><br>Partners in the practice and employed veterinarians. Range of experience from 1 year (interns) to over 35 years graduated.	3
C	Independently owned mixed practice. RCVS accredited Equine Veterinary Hospital	50	14	10 (6 female, 4 male)<br><br>Partners in the practice and employed veterinarians. Some treated horses only, some treated horses as well as companion and production species. Range of experience from 1 year (interns) to over 30 years graduated.	8 (1 written)
D	Independently owned, RCVS accredited Equine General Practice	66	10	7 (6 female, 1 male)<br><br>Partners in the practice and employed veterinarians. Range of experience from 2 to 23 years graduated.	10

S4. Owner interview participant details

Practice	Consultation type (as presented)	Horse housing premises	Horse details
A	Follow up consultation for steroid injection	DIY livery	28-year-old Cob gelding. Previously used by owner for driving and mixed leisure pursuits, stopped riding last 5 months
A	Sedate for farrier, check sarcoid and additional pony presented during visit for pain and hoof abnormality	DIY livery	9 horses in total. Hacking and carriage driving
A	Lameness and throwing head up	DIY livery	14-year-old mare. Also owns a 21-year-old mare as well as donkeys
A	Vaccination	DIY livery	17-year-old gelding, 10-year-old gelding and previously owned a 30-year-old gelding
B	Admit horse to the hospital for stifle injection	Reported to be kept on a farm near house	20-year-old Thoroughbred cross, gelding. Hunter, being brought back into work over the autumn
B	Lameness	Private home premises	7-year-old and 12-year-old geldings. Both used for eventing
B	Review intermittent lameness	DIY livery	7-year-old Irish Sports Horse, gelding. Leisure/pleasure
C	Put to sleep (euthanasia)	DIY field and stables for 3 horses	Cob, mare. Companion horse. Also owned a 7-year-old retired gelding and 2 horses kept at another premises
C	Dental and blood tests (Adrenocorticotropic hormone/ACTH)	Rented stables and fields at farm near to home	25-year-old Connemara gelding
C	Blood tests (ACTH)	DIY livery	21-year-old Thoroughbred cross, gelding. Leisure/pleasure. Also own another horse
C	Lameness	DIY livery	24-year-old Welsh Cob mare. Leisure/pleasure
C	Vaccination and two prescription check-ups	Large DIY livery	21-year-old Standardbred gelding, retired, 26-year-old mare and 13-year-old mare
C	Presented at hospital for radiographs for laminitis review	Kept at own home with companion pony	10-year-old Connemara cross, mare. Pony club competition

C	Presented at hospital for 'MOT'	Farm livery	9-year-old Thoroughbred cross, gelding. Eventing
C	Behaviour changes when ridden	Large DIY livery	8-year-old sports horse, mare. Leisure/pleasure<br>(Written interview responses)
C	Presented at hospital. Re-examine post kissing spine surgery	Large DIY livery	10-year-old warmblood, gelding. Dressage, show jumping, hacking. Previously owned an 18-year-old Irish Sports Horse, gelding
D	Re-scan ligament	DIY livery	13-year-old Welsh Cob, mare. Leisure, hacking
D	Ridden assessment/performance review for potential lameness	DIY livery<br>(2 owners and livery yard owner interviewed)	6-year-old mare, backed last year. Hacking, school work. Also own another retired gelding
D	Prescription check-up and sedate for dental	Private rented stables and field	28-year-old Irish Sports Horse, gelding and 11-year-old thoroughbred cross gelding. Leisure, hacking
D	Re-scan following tendon injury	DIY livery	10-year-old Thoroughbred, gelding. Leisure, hacking
D	Radiographs following emergency veterinary visit	Kept at own home	34-year-old Arab, gelding. Retired
D	Lameness investigation (clinic)	Home premises	10-year-old gelding. Eventing
D	Medicate hock and back	Assisted livery	17-year-old Irish Draught cross warmblood, gelding. Hacking
D	Re-examine for stifle injection, second lame horse, vaccinate 7 horses	Riding centre for non-able-bodied riders	Multiple horses owned, generally reported to be older animals
